# Integrative Metagenomic Analyses Reveal Gut Microbiota-Derived Multiple Hits Connected to Development of Diabetes Mellitus

**DOI:** 10.3390/metabo14120720

**Published:** 2024-12-21

**Authors:** Sehad N. Alarifi, Essam Jamil Alyamani, Mohammed Alarawi, Azzam A. Alquait, Mohammed A. Alolayan, Ahmad M. Aldossary, Randa A. Abd EL-Rahman, Rashid Mir

**Affiliations:** 1 Departments of Food and Nutrition Science, Al-Quwayiyah College of Sciences and Humanities, Shaqra University, Al-Quwayiyah 11971, Saudi Arabia; 2 Wellness & Preventive Medicine Institute Health Sector King Abdulaziz City for Science and Technology (KACST), Riyadh 11442, Saudi Arabia; eyamani@kacst.edu.sa (E.J.A.); aalqait@kacst.gov.sa (A.A.A.); aaldossary@kacst.gov.sa (A.M.A.); 3 King Abdullah University of Science and Technology (KAUST), Jeddah 23955, Saudi Arabia; 4 Department of Biology, Al-Quwayiyah College of Sciences and Humanities, Shaqra University, Riyadh 11971, Saudi Arabia; randa@su.edu.sa; 5 Department of Medical Lab Technology, Faculty of Applied Medical Sciences, Prince Fahad Bin Sultan Chair for Biomedical Research, University of Tabuk, Tabuk 71491, Saudi Arabia; rashid@ut.edu.sa

**Keywords:** metagenomics, type 2 diabetes, T2DM, gut microbiota dysbiosis, function of gut microbiota, gut microbiota, gut microbiome, 16S rRNA sequencing, microbial composition, metabolic disorder

## Abstract

Background/Objectives: Type 2 diabetes mellitus (T2DM) is a chronic metabolic disorder associated with gut dysbiosis. To investigate the association between gut microbiota and T2DM in a Saudi Arabian population. Methods: We conducted a comparative analysis of fecal microbiota from 35 individuals, including both T2DM patients and healthy controls. 16S rRNA gene sequencing was employed to characterize the microbial community structure. Results: Our findings revealed significant differences in microbial composition between the two groups. The T2DM group exhibited a higher abundance of Firmicutes and lower levels of Bacteroidetes compared to the healthy control group. At the genus level, T2DM patients showed a decrease in butyrate-producing bacteria such as Bacteroides and Akkermansia, while an increase in Ruminococcus and Prevotella was observed. Additionally, the T2DM group had a higher abundance of Faecalibacterium, Agathobacter, and Lachnospiraceae, along with a lower abundance of Bacteroides. Conclusions: These results suggest that alterations in gut microbiota composition may contribute to the development of T2DM in the Saudi Arabian population. Further large-scale studies are needed to validate these findings and explore potential therapeutic interventions targeting the gut microbiome.

## 1. Introduction

Type 2 diabetes mellitus (T2DM) is a significant global health concern affecting millions worldwide [1]. Characterized by hyperglycemia, insulin resistance, and impaired insulin production, T2DM is influenced by various factors, including genetics, lifestyle, and dietary habits [2,3]. Recent research has increasingly recognized the role of gut microbiota in the development of T2DM [4,5,6,7].

Saudi Arabia, with its rapid lifestyle and dietary changes, has witnessed a significant rise in T2DM prevalence, ranking among the top 10 nations globally [8,9]. This trend is alarming, given the potential for severe health complications and reduced life expectancy associated with poorly controlled diabetes [10,11]. Studies from Saudi Arabia have reported suboptimal HbA1c control in a substantial proportion of T2DM patients, highlighting the need for effective management strategies [12,13,14,15].

The gut microbiota, a complex community of microorganisms, plays a crucial role in maintaining overall health. Alterations in gut microbiota composition, known as dysbiosis, have been linked to various metabolic disorders, including T2DM [4]. These microbial shifts can impact gut permeability, inflammation, and metabolic regulation. Additionally, the gut microbiota interacts with host metabolism by influencing the secretion of gut hormones, glucose regulation, and lipid metabolism. Previous studies have utilized 16S rRNA and whole metagenome shotgun sequencing to investigate the composition and function of gut microbial communities in prediabetic individuals [16].

While recent research has highlighted the potential role of gut microbiome dysbiosis in the development and progression of T2DM [17,18], conflicting findings exist regarding the specific microbial alterations associated with the disease. Some studies have reported decreased levels of Bacteroidetes and increased levels of Firmicutes in individuals with T2DM [5,7,19], while others have found contrasting results [4]. Furthermore, geographical variations, dietary habits, and medication use can influence gut microbiota composition and complicate the identification of a universal T2DM-associated microbiome profile [20,21,22,23].

Given the increasing prevalence of T2DM in Saudi Arabia and the potential impact of gut microbiota on disease development, this work aimed to investigate the diversity and composition of the gut microbiome in Saudi individuals with and without T2DM using metagenomic sequencing. By understanding the specific microbial alterations associated with T2DM in this population, we can potentially identify novel therapeutic targets and preventive strategies to combat this growing health crisis.

## 2. Material and Methods

### 2.1. Study Populations

The study was conducted after obtaining ethical approval from the Shaqra University Ethics Research Committee (ERC_SU_20220053) dated 20 June 2022. and in accordance with the Helsinki Declaration. A total of 24 Saudi individuals diagnosed with type 2 diabetes mellitus (T2DM) were randomly selected from the endocrinology clinic. Additionally, 11 healthy controls with HbA1c levels below 6% were included in the study. All participants were provided with detailed information about the study objectives, design, and confidentiality measures. Written informed consent was obtained from each participant. Sterile stool specimen containers with integrated collection spoons and instructions were provided to all participants. The recruitment process extended over a six-month period from May to October 2023.

Basic demographic characteristics, including gender, height, age, weight, and body mass index (BMI), was recorded for each participant. [Table 1] Blood samples were collected to analyze HbA1c levels, total cholesterol, and vitamin D levels. Stool samples were collected immediately and stored at −80 °C.

To be eligible for the study, participants had to be 18 years of age or older. Individuals who had received antibiotic treatment within the past three months, were pregnant or lactating, or had inflammatory bowel disease were excluded from the study.

### 2.2. Sample Collection

A total of 35 blood and fecal samples were collected from diabetic patients. To ensure sample integrity, stringent sterile conditions were maintained throughout the collection process. Subsequently, the samples were stored at a temperature of −80 °C until further analysis, which included 16S rRNA gene sequencing and whole metagenome sequencing.

### 2.3. DNA Library Preparation and Sequencing

Fecal samples were subjected to DNA extraction using the QIAamp PowerFecal DNA Kit, adhering to the manufacturer’s protocol (Qiagen GmbH, Hilden, Germany). The extracted DNA was subsequently stored at −80 °C for future analyses.

The V4 region of the 16S rRNA gene was amplified using the primer pair 515F (GTGCCAGCMGCCGCGGTAA) and 806R (GGACTACHVGGGTWTCTAAT) [24]. PCR amplification of the targeted regions was performed using specific primers linked to unique barcodes. Amplicons of the appropriate size were selected through 2% agarose gel electrophoresis.

Equal amounts of PCR products from each sample were pooled, followed by end-repair, A-tailing, and ligation with Illumina adapters. The resulting libraries were sequenced on an Illumina platform, generating 250 base pair paired-end raw reads. Before sequencing, the library quality was assessed using Qubit for quantification and a Bioanalyzer for size distribution analysis. Quantified libraries were pooled and sequenced on Illumina platforms based on the desired sequencing depth and library concentration.

### 2.4. Bioinformatics Analysis

Paired-end reads were initially sorted based on their unique barcodes and then trimmed to remove barcode and primer sequences. These paired-end reads were merged into raw tags using Fast Length Adjustment of SHort reads, FLASH software (32.0.0.142) [25]. To ensure high-quality data, the raw tags were subjected to quality filtering using Fastp software (Version 0.23.1) [26]. The resulting high-quality clean tags were compared against reference databases (Silva for 16S/18S rRNA genes and Unite for ITS (Internal transcribed spacer) sequences) to identify and remove chimeric sequences using Vsearch software- A versatile open source tool for metagenomics (V2.16.0) [27]. 

The effective tags were then denoised using either DADA2 or Deblur within the QIIME2 software package (Version QIIME2-202202) to generate amplicon sequence variants (ASVs). Taxonomic annotation was performed using QIIME2, with the Silva database (https://www.arb-silva.de/, accessed on 11 December 2024) for 16S/18S sequences and the Unite database (https://unite.ut.ee/, accessed on 11 December 2024) for ITS sequences. Multiple sequence alignment was carried out using QIIME2 to study the phylogenetic relationships between ASVs and to identify differences in dominant species across different samples or groups.

**Internal transcribed spacer sequences (ITS).** Multiple sequence alignment was carried out using QIIME2 to study the phylogenetic relationships between ASVs and to identify differences in dominant species across different samples or groups.

Species across different samples or groups.

To account for variations in sequencing depth, the absolute abundance of ASVs was normalized based on the sample with the lowest number of sequences. Alpha diversity analysis was performed using QIIME2 to assess species richness and evenness within each sample. Beta diversity analysis, calculated using weighted and unweighted UniFrac distances in QIIME2, was used to compare community composition differences between samples or groups. Weighted UniFrac considers both the presence and abundance of species, while unweighted UniFrac focuses solely on the presence/absence of species.

### 2.5. Statistical Analysis

All statistical analyses were performed using R (version 4.0.3). To investigate the differentiation of community structure, a series of statistical analyses were conducted, including *t*-test and Wilcoxon test. To predict metagenomic functions based on marker genes, PICRUSt2 (version 2.3.0) was employed. Differential metabolic pathway analysis was performed using the *t*-test.

## 3. Results

### 3.1. Basic Characteristics of the Study Cohort

Basic demographic information, including age, gender, height, weight, and body mass index (BMI), was recorded for each participant. [Table 1] Blood samples were withdrawn to analyze HbA1c levels, total cholesterol, and vitamin D levels. 

A total of 24 individuals with type 2 diabetes mellitus (T2DM) and 11 healthy controls were enrolled in this study. A significant difference was observed in the gender distribution between the two groups. The diabetic group was predominantly male (79%), while the healthy group was predominantly female (64%) (Fisher’s exact test, *p* < 0.05). Additionally, a significant age difference was noted between the two groups. The median age in the diabetic group was 55 years (IQR: 47–59), whereas the median age in the healthy group was 28 years (IQR: 25–30) (Wilcoxon test, *p* < 0.001). The majority of diabetic patients were aged between 40 and 60 years (74%), with 8% aged 20–40 and 21% aged over 60. In contrast, all healthy controls were aged between 20 and 40 years. Regarding glycemic control, 54% of the diabetic group had HbA1c levels between 6.5% and 8.1%, indicating fair control. However, 38% of the diabetic group had poorly controlled HbA1c levels (>8.1%). Among the ten healthy controls with available HbA1c information, nine had levels below 5.7%, indicating optimal control, and one had a prediabetic level. Cholesterol levels were mostly within the normal range in both groups and did not differ significantly. A small proportion of individuals in both groups had borderline high cholesterol (25% in the diabetic group and 30% in the healthy group). However, no individuals in the healthy group had high cholesterol, while 13% of the diabetic group did. Vitamin D deficiency was observed in 13% of the diabetic group, insufficiency in 39%, and sufficiency in 48%. In the healthy group, 80% had vitamin D insufficiency, and 20% had sufficiency. Regarding body weight, only 10% of the diabetic group and 20% of the healthy group were classified as normal weight. Overweight individuals accounted for 50% and 40% of the diabetic and healthy groups, respectively. Additionally, 40% of the diabetic group and 30% of the healthy group were classified as obese.

### 3.2. Relative and Taxonomic Abundance Among Samples

To further investigate the microbial composition within each T2DM and healthy sample, a detailed analysis of the most abundant phyla was conducted. Bacteroidota and Firmicutes were the two predominant phyla observed across all samples. However, some variations were noted. One T2DM sample exhibited a dominance of Euryarchaeota, while one healthy sample showed a prevalence of Verrucomicrobiota.

**Figure 1:**
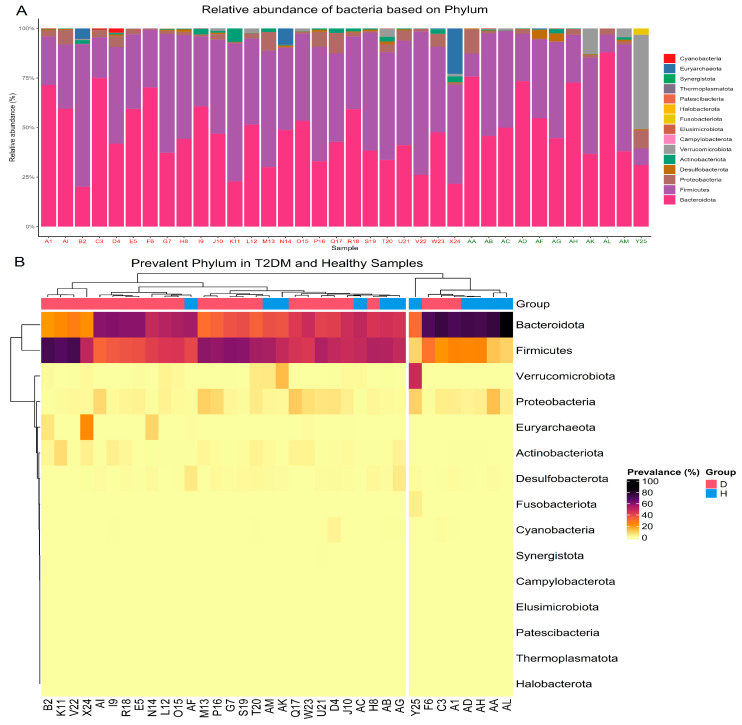
(**A**) Relative abundance of the top phyla in T2DM and healthy individuals. X-axis represents the diabetes samples in red and healthy samples in green. Y-axis represents relative abundance as a percentage. (**B**) Heatmap illustrating the distribution of top phyla across samples, with hierarchical clustering applied to both samples and phyla. Columns represent the sample while rows represent phyla.

Among the T2DM samples, nine showed a Bacteroidota prevalence of over 50%, while five samples had a prevalence of less than or equal to 30%. The remaining T2DM samples exhibited a Bacteroidota prevalence between 30% and 50%. In contrast, ten T2DM samples showed a Firmicutes prevalence of over 50%, three samples showed a prevalence of less than or equal to 30%, and eleven samples showed a prevalence between 30% and 50%. In the healthy group, eight out of eleven samples showed a Bacteroidota prevalence of over 45%, and five samples showed a *Firmicutes* prevalence of over 45%. Notably, Euryarchaeota was predominantly present only in T2DM samples, while Verrucomicrobiota was predominantly found in healthy samples. Figure 1A illustrates the predominant phyla in all the studied samples.

To further explore the relationships between samples based on their microbial composition, unsupervised hierarchical clustering was performed using the top phyla. This analysis resulted in the formation of two main clusters. One cluster predominantly contained T2DM samples, characterized by a dominance of Firmicutes or a shared dominance with Bacteroidota. The other cluster primarily included healthy samples, with Bacteroidota as the dominant phylum. Figure 1B depicts the heatmap and clustered samples.

Overall, the results suggest that T2DM samples exhibit greater microbial diversity compared to healthy samples, with a noticeable shift in dominance from Bacteroidota to Firmicutes.

### 3.3. Microbial Compositional Differences Between T2DM and Healthy Individuals

**Figure 2:**
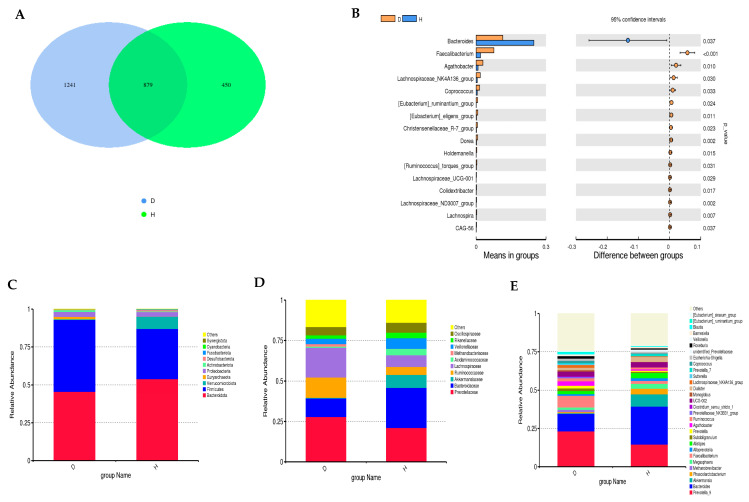
(**A**) Venn diagram depicting shared and unique taxa between T2DM and healthy individuals. (**B**) Differential abundance analysis of specific genera between T2DM and healthy groups. The left panel presents the bar graphs depicting mean value of the abundance of the genus in each group. The right panel is the confidence interval of between-group variations. The right-most value is the *p*-value of the significance test of between-group variation using the *t*-test. (**C**–**E**) Comparative analysis of the relative abundance of top phyla, families, and genera between T2DM and healthy groups.

To further elucidate the microbial differences between T2DM and healthy individuals, a comparative analysis was conducted at the phylum, class, family, and genus levels. A total of 879 taxa were shared between the two groups. However, the T2DM group exhibited a higher degree of microbial diversity, with 1241 unique taxa not found in healthy individuals. Conversely, the healthy group had 450 unique taxa (**Figure 2A**).

At the phylum level, Bacteroidetes and Firmicutes were the dominant phyla in both groups. However, their relative abundances differed significantly. Healthy individuals displayed a higher proportion of Bacteroidetes, while diabetic patients had a higher abundance of Firmicutes. At the class level, Bacteroidia was more prevalent in healthy individuals, whereas Clostridia was more abundant in diabetic patients. Additionally, the Methanobacteria class was specific to the diabetic group, while Negativicutes and Verrucomicrobiae classes were more prominent in the healthy group (**Figure 2B**).

At the family level, *Bacteroidaceae* was more abundant in healthy individuals, while *Prevotellaceae* was more prevalent in the diabetic group. Notably, *Lachnospiraceae* and *Ruminococcaceae* were enriched in the diabetic group, whereas *Akkermansiaceae* was more abundant in the healthy group. At the genus level, *Bacteroides* was dominant in the healthy group, along with *Akkermansia* and *Alistipes*. In contrast, *Prevotella_9* was the most abundant genus in the diabetic group. A statistical comparison between the T2DM and healthy groups revealed significant differences in the abundance of specific microbial genera. *Faecalibacterium* was significantly enriched in the T2DM group compared to the healthy group (*t*-test; *p* < 0.001) (Figure 2**C**–**E**). Additionally, Genus *Agathobacter* and *Coprococcus* showed increased abundance in the T2DM group (*t*-test; *p* < 0.05). Conversely, a decrease in the abundance of *Bacteroides* was observed in the T2DM group compared to the healthy group (*t*-test; *p* < 0.05).

### 3.4. Microbial Alpha-Diversity Comparative Analysis

Alpha diversity metrics were employed to assess the richness and evenness of microbial communities within each sample. A series of statistical analyses, including Chao1, dominance, observed features, Pielou’s evenness, Shannon, and Simpson indices, were performed. Table 2 represents the summary of these indices in this cohort.

A comparison of these indices between the T2DM and healthy groups revealed significant differences. The Chao1 index, which estimates species richness, was significantly higher in the T2DM group compared to the healthy group (Welch’s two-sample *t*-test, *p* = 0.038). Similarly, the number of observed features was significantly higher in the T2DM group (Welch’s two-sample *t*-test, *p* = 0.038). The Shannon index, which considers both species richness and evenness, was also higher in the T2DM group (*p* = 0.033). Pielou’s evenness index, which measures community evenness, showed a trend towards higher values in the T2DM group but was not significant (*p* = 0.058). However, no significant differences were observed in the dominance and Simpson indices between the two groups. These findings suggest that the T2DM group exhibits higher microbial diversity compared to the healthy group.

### 3.5. Microbial Beta Diversity Compararative Analysis

**Figure 3:**
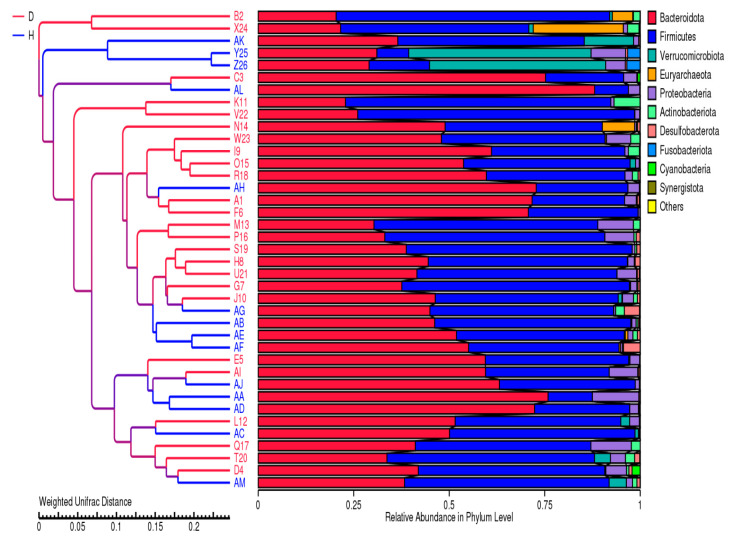
UPGMA clustering of samples based on weighted UniFrac distance, with the relative abundance of phyla visualized for each sample. The phylogenetic tree shows the distances between samples, with T2DM samples marked in red and healthy samples in blue. The accompanying bar chart displays the abundance of microbial taxa, highlighting the dominance of phyla.

**Figure 4:**
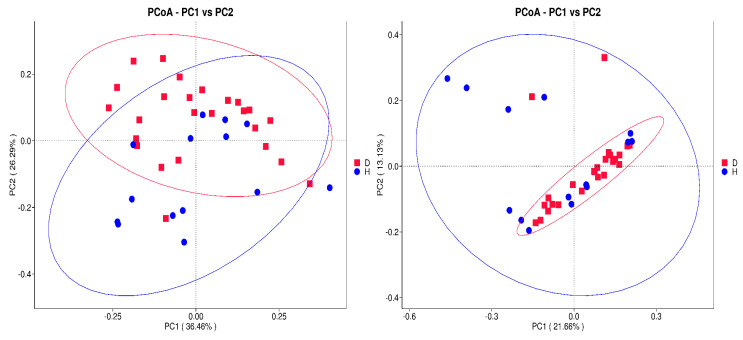
Principal coordinate analysis (PCoA) plots based on weighted and unweighted UniFrac distances, illustrating the separation between T2DM and healthy samples. Each point on the plot represents a sample, positioned according to one principal component on the X-axis and another on the Y-axis. The points are color-coded by group. The percentage shown on each axis indicates the contribution of that principal component to the overall variance among the samples.

Beta diversity analysis was employed to assess the compositional differences in microbial communities between T2DM and healthy individuals. To this end, unweighted and weighted UniFrac distances were calculated based on phylogenetic relationships and abundance information of microbial features.Principal coordinate analysis (PCoA) and unweighted pair group method with arithmetic mean (UPGMA) clustering was performed to visualize and analyze inter-group differences. The results consistently demonstrated a clear separation between T2DM and healthy samples. The T2DM samples formed distinct clusters, while the healthy samples clustered together.Furthermore, the relative abundance of phyla was analyzed. Firmicutes and Bacteroidota were identified as the most prevalent phyla, with a few samples exhibiting higher levels of Verrucomicrobiota followed by Proteobacteria. The T2DM-enriched clusters were characterized by a predominance of Firmicutes, while the healthy clusters were dominated by Bacteroidota and Verrucomicrobiota. Figure 3 and Figure 4 depicts the UPGMA clustering and PCoA analysis respectively.

These findings strongly suggest that T2DM and healthy individuals harbor distinct microbial community structures, with significant differences in both phylogenetic relationships and microbial abundance.

### 3.6. Functional Changes in Gut Microbiome

**Figure 5:**
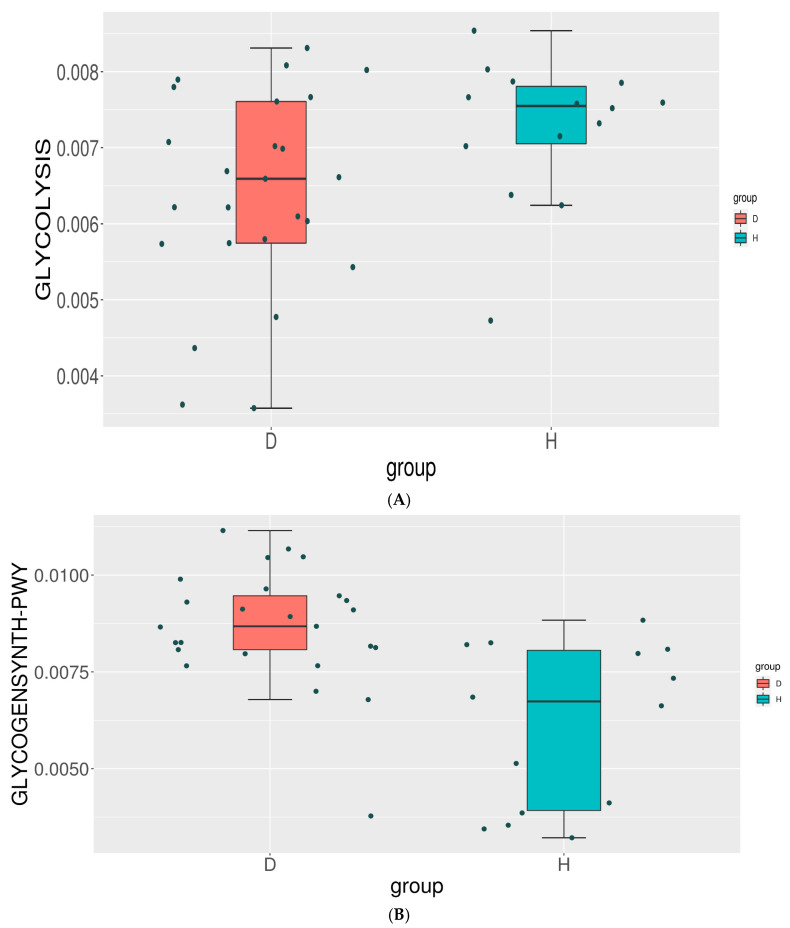

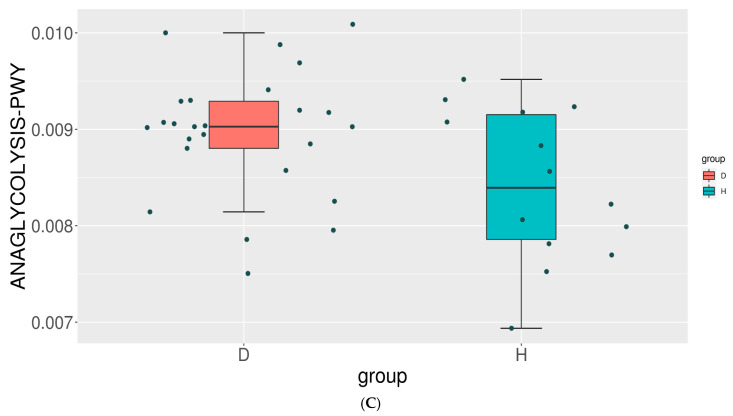
Box plots comparing the relative abundance of specific metabolic pathways between T2DM and healthy individuals: (**A**) glycogen synthesis pathway; (**B**) glycolysis pathway; and (**C**) anaerobic glycolysis pathway.

To predict the functional potential of the microbial communities, PICRUSt analysis was employed, utilizing 16S rRNA gene sequence data and the Kyoto Encyclopedia of Genes and Genomes (KEGG) database. The top 35 most abundant functions were identified for each sample. A comparative analysis of these predicted functions revealed significant differences between T2DM and healthy individuals. The glycolysis pathway, responsible for breaking down glucose into pyruvate and short-chain fatty acids (SCFAs), was predicted to be more active in the healthy group. SCFAs play a crucial role in gut health and insulin sensitivity. Conversely, the glycogen synthesis pathway, which converts glucose into glycogen, was predicted to be more active in the T2DM group. This pathway may contribute to altered glucose metabolism and insulin resistance in T2DM. Additionally, the anaerobic glycolysis pathway was predicted to be slightly more active in the T2DM group. Figure 5 illustrates the differential metabolic pathways between the two groups.

## 4. Discussion

The primary aim of this pilot study was to explore the gut microbiota and associated bacteria in T2DM patients, compared to healthy subjects from a rural community in Saudi Arabia, where the traditional diet excludes Western food. Our findings indicate that the gut microbiota of T2DM is distinct from that of healthy controls. Both groups showed a predominance of Firmicutes and Bacteroidetes, in line with findings from Emirati populations [28]. However, the relative abundance of *Firmicutes* was higher in T2DM patients, whereas the healthy individuals showed a greater proportion of Bacteroidetes and Verrucomicrobiota. This is consistent with previous studies suggesting that the Firmicutes/Bacteroidetes ratio is higher in T2DM [7,21,28]. A study from Saudi Arabia also confirmed this pattern, with Firmicutes being prevalent in T2DM patients, along with increased microbial diversity in patients with higher HbA1c [19,29].

Our observations differ from the studies conducted in Western countries, where lower Firmicutes and Clostridia were reported to be lower in diabetic individuals [4]. On the other hand, research on African populations indicated that, in comparison with other populations, Firmicutes, Actinobacteria, Bacteroidetes, *Clostridiaceae*, and *Peptostreptococcaceaea* were notably reduced in T2DM patients [30]. In an Indian study, Firmicutes, Bacteroidetes, Proteobacteria and Actinobacteria were not significantly different from those in healthy controls [31].

Studies have confirmed the importance of metformin in regulating gut microbiota [32,33]. Although our study did not specifically assess metformin usage, its effects could be inferred based on previous literature [5,34,35]. A strong relationship has been observed between differences in ancestry, geographic regions, eating habits, research methods and gut microbiota [36]. Another possible explanation for this result is that, due to age, the diabetic group as more likely to be elderly, while the non-diabetic consisted of young adults; hence, microbial composition homeostasis mayt be subject to change [37].

Another finding at the class level was that Clostridia, and at the family level *Lachnospiraceae* and *Ruminococcaceae*, belonging to Firmicutes phylum, as well as the order level Oscillospirales and Lachnospirales, were abundant in diabetes patients. This could be explained by a non-Western, unprocessed diet. In this study, we did not include dietary intake due to insufficient data. The abundance of these families is associated with a healthy lifestyle and healthy diet due to their ability to digest complex plant materials that are indigestible by the host [38]. Other study shows an increase in both families following a 100% plant-based diet intervention combined with exercise and stress management, suggesting that changes in lifestyle and diet may effect their richness [39]. *Lachnospiraceae* and *Ruminococcaceae* have been found in diabetes patients in a previous study conducted in China [40]. *Oscillospirales* genus *Oscillospia* was associated positively with development and inflammation in T2DM disease in animal study [41].

Diabetes was associated positively with *Lachnospiraceae*, *Faecalibacterium*, *and Agathobacter*, and negatively with *Bacteriodes*. *Fecalbacterium* was inversely associated with fasting glucose in previous studies [42]. Another study on Mexican pediatric patients with metabolic syndrome and diabetes found a significant association between glucose levels, obesity, and *Faecalibacterium* [43,44]. Remarkably, in this study, this bacterium was positively associated with the diabetes group This could be explained by antidiabetic medications, notably Metformin, which have been shown to exert an important effect on the metagenomic profiles of individuals with T2DM [6,34].

The present findings indicate an increased relative abundance of *Prevotellaceae_9* genera in individuals with T2D. This increased abundance is consistent with a UAE study that similarly reported a higher presence of *Prevotella* among individuals with T2DM [21]. This study suggests that this bacterial group tends to appear in diabetic individuals but is less prevalent in healthy populations. This distinction highlights its potential as a microbial marker specific to diabetes. Additionally, previous studies have linked *Prevotellaceae_9* to varying levels of diabetes complications, suggesting its possible role in predicting the progression or severity of the disease [45]. On the other hand, an earlier study in the UAE found a decrease in this genus in T2DM individuals [22]. The association of this genus with dietary fiber and polysaccharide metabolism implies that the dietary habits of the T2DM group, particularly in rural regions, could be characterized by higher fiber intake and traditional, non-Western foods such as whole wheat [46,47]. Notably, a previous study linked the increased presence of *Prevotella* with poorer glycemic control [48]. Overall, these results may indicate an alteration in gut microbiota related to glucose levels and complications of diabetes, and thus can support research into diagnosing the stages of development of this disease.

Healthy controls exhibited an increased presence of *Akkermansia*, *Bacteroides*, and *Alistipes* genera, consistent with previous studies conducted in China on diabetic individuals [40,48]. T2DM patients display significantly lower levels of *Akkermansia muciniphila*, which has been shown to regulate intestinal permeability by preserving the mucosal layer, which in turn is associated with glycemic control, insulin resistance management, and lower plasma cholesterol [49]. *Akkermansia* is vital for maintaining the integrity of the mucin layer and reducing inflammation. Mucins are large, highly glycosylated proteins that play a role in the luminal protection of the GIT, leading to a reduction in bacterial translocation and improvements in the storage of fat, adipose tissue metabolism, and glucose homeostasis [50].

Regarding the functional differences in gut microbiota between the two groups, the glycolysis pathway was found in healthy controls, responsible for breaking down carbohydrates and producing short-chain fatty acids such as acetate, propionate, and butyrate, which contribute to host health. On the other hand, glycogenesis was observed in the disease group, which is responsible for glycogen synthesis. This finding indicates that SCFAs may be involved in T2DM development. Among them, butyrate is one of the most well-studied SCFAs produced by the gut microbiota. It could ameliorate the progression of T2DM via a variety of mechanisms, including maintaining the integrity of the intestinal epithelial barrier [51], promoting liver glycogen metabolism [52], and regulating the function of mitochondria [53]. Likewise, acute supplementation of propionate increased postprandial plasma levels of these two gut hormones in humans, and its long-term supplementation prevented weight gain and worsening insulin sensitivity in overweight adults [54]. Moreover, propionate activated intestinal gluconeogenesis via the GPR41-dependent gut-brain neural circuit, thereby maintaining glucose and energy homeostasis [55].

## 5. Strength

This is the first study investigating a Saudi rural, non-Western diet population. Overall, our results emphasize the previous studies suggestion, which is that different taxa are indeed reported to be associated with T2DM positively and negatively in different studies. We confirm the differences between T2DM and HC in the abundance of several different genera.

## 6. Limitation

This pilot study focused on limited numbers of Saudi rural area participants only, so the results and interpretation should be taken with caution. The recruitment of volunteers was very challenging due to people’s reluctance to give stool samples. The lower number of T2DM patients and healthy controls could be one explanation for richness of the disease group microbiota. Differences in diet may be another factor affecting the results, as no detailed description of the diet was recorded. To strengthen the foundation for developing bacterial signatures for T2D, future studies should be larger, international, multi-site studies, to account for variation in inter-individual microbiota. Possible confounding factors that ought to be controlled closely include medication use, diet, and lifestyle.

## 7. Conclusions

In conclusion, this pilot study revealed that Saudi diabetes patients have a distinct microbiome composition compared to the healthy control population at the phylum, family, and genus levels. Additionally, T2DM glucose levels and the development of the disease result in differences in the diversity features of intestinal microorganisms. This may contribute to the complex interaction between the host and the gut microbiota.

## Figures and Tables

**Table 1:** Clinical and demographic characteristics of Saudi Arabian T2DM cases (n = 24) and controls (n = 11).

Characteristic	Diabetes	Healthy	*p*-Value ^2^
	N = 24 ^1^	N = 11 ^1^	
**Gender**			**0.022**
Female	5 (21%)	7 (64%)	
Male	19 (79%)	4 (36%)	
**Age (Years)**	55 (47, 59)	28 (25, 30)	**<0.001**
**Age Group**			**<0.001**
20–40	2 (8.3%)	11 (100%)	
40–60	17 (71%)	0 (0%)	
>60	5 (21%)	0 (0%)	
**HBA1c**			**<0.001**
Normal (<5.7%)	1 (4.2%)	9 (90%)	
Pre-Diabetic (5.7–6.4%)	1 (4.2%)	1 (10%)	
Diabetic (6.5–8.1%)	13 (54%)	0 (0%)	
Poorly Controlled Diabetic (>8.1%)	9 (38%)	0 (0%)	
**Total cholesterol (mmol/L)**			0.7
Normal (<5.2)	15 (63%)	7 (70%)	
Borderline (5.2–6.2)	6 (25%)	3 (30%)	
High (>6.2)	3 (13%)	0 (0%)	
**Vitamin D (ng/mL)**			0.1
Deficiency (<10)	3 (13%)	0 (0%)	
Insufficiency (10–29)	9 (39%)	8 (80%)	
Sufficiency (≥30)	11 (48%)	2 (20%)	
**BMI (kg/m^2^)**			0.5
Underweight (<18.5)	0 (0%)	1 (10%)	
Normal (18.5–24.9)	2 (10%)	2 (20%)	
Overweight (25–29.9)	10 (50%)	4 (40%)	
Obese (≥30)	8 (40%)	3 (30%)	

^1^ n (%); median (IQR) ^2^ Fisher’s exact test; Wilcoxon rank sum test.

**Table 2:** Mean value of different alpha diversity indices in diabetes vs. healthy group. All the significant *p*-values are highlighted in bold.

Index	Diabetes	Healthy	*p*-Value ^2^
	N = 24 ^1^	N = 11 ^1^	
Chao1	331 (90)	244 (118)	0.038
Dominance	0.08 (0.06)	0.11 (0.07)	0.2
Observed Features	330 (89)	243 (118)	0.038
Pielou’s evenness	0.63 (0.08)	0.58 (0.06)	0.058
Shannon	5.23 (0.79)	4.52 (0.91)	0.033
Simpson	0.92 (0.06)	0.89 (0.07)	0.2

^1^ Mean (SD); ^2^ Welch’s two-sample *t*-test.

## Data Availability

All data is available on request at. Departments of Food and Nutrition Science, Al-Quwayiyah College of Sciences and Humanities, Shaqra University, Al-Quwayiyah 11971, Saudi Arabia. snalarifi@su.edu.sa.
